# Mutational spectrum of breast cancer susceptibility genes among women ascertained in a cancer risk clinic in Northeast Brazil

**DOI:** 10.1007/s10549-022-06560-0

**Published:** 2022-03-30

**Authors:** Gabriela E. S. Felix, Rodrigo Santa Cruz Guindalini, Yonglan Zheng, Tom Walsh, Elisabeth Sveen, Taisa Manuela Machado Lopes, Juliana Côrtes, Jing Zhang, Polyanna Carôzo, Irlânia Santos, Thaís Ferreira Bonfim, Bernardo Garicochea, Maria Betânia Pereira Toralles, Roberto Meyer, Eduardo Martins Netto, Kiyoko Abe-Sandes, Mary-Claire King, Ivana Lucia de Oliveira Nascimento, Olufunmilayo I. Olopade

**Affiliations:** 1 Instituto de Ciências da Saúde, Universidade Federal da Bahia, Salvador, Bahia Brazil; 2 Centro de Pesquisas Gonçalo Moniz, Fundação Oswaldo Cruz Bahia, Salvador, Bahia Brazil; 3 Centro de Investigação Translacional em Oncologia (CTO), Instituto do Cancer do Estado de São Paulo (ICESP), Hospital das Clínicas da Faculdade de Medicina da Universidade de São Paulo, São Paulo, São Paulo Brazil; 4 Instituto D’or de Pesquisa e Ensino, Salvador, Bahia Brazil; 5 Department of Medicine, Center for Clinical Cancer Genetics and Global Health, University of Chicago, Chicago, Illinois USA; 6 Division of Medical Genetics, Department of Medicine, University of Washington, Seattle, Washington USA; 7 Universidade do Estado da Bahia, Salvador, Bahia Brazil; 8 Grupo Oncoclínicas, São Paulo, São Paulo Brazil; 9 Laboratório de Pesquisa em Infectologia, Hospital Universitário Prof. Edgard Santos, Universidade Federal da Bahia, Salvador, Bahia Brazil; 10 Núcleo de Oncologia da Bahia, Salvador, Bahia Brazil; 11 Department of Medicine, Center for Clinical Cancer Genetics and Global Health, University of Chicago, 5841 S. Maryland Avenue, MC 2115, Chicago, Illinois 60637-1470 USA

**Keywords:** Breast cancer, Brazilians, BROCA panel, Genetic testing

## Abstract

**Purpose:**

There is a paucity of data on the spectrum and prevalence of pathogenic variants among women of African ancestry in the Northeast region of Brazil.

**Methods:**

We performed BROCA panel sequencing to identify inherited loss-of-function variants in breast cancer susceptibility genes among 292 Brazilian women referred to a single institution cancer risk assessment program.

**Results:**

The study included a convenient cohort of 173 women with invasive breast cancer (cases) and 119 women who were cancer-free at the time of ascertainment. The majority of the women self-reported as African-descended (67% for cases and 90.8% for unaffected volunteers). Thirty-seven pathogenic variants were found in 36 (20.8%) patients. While the spectrum of pathogenic variants was heterogeneous, the majority (70.3%) of the pathogenic variants were detected in high-risk genes *BRCA1*, *BRCA2*, *PALB2*, and *TP53*. Pathogenic variants were also found in the *ATM*, *BARD1*, *BRIP1*, *FAM175A*, *FANCM*, *NBN*, and *SLX4* genes in 6.4% of the affected women. Four recurrent pathogenic variants were detected in 11 patients of African ancestry. Only one unaffected woman had a pathogenic variant in the *RAD51C* gene. Different risk assessment models examined performed well in predicting risk of carrying germline loss-of-function variants in *BRCA1* and/or *BRCA2* in breast cancer cases.

**Conclusion:**

The high prevalence and heterogenous spectrum of pathogenic variants identified among self-reported African descendants in Northeast Brazil is consistent with studies in other African ancestry populations with a high burden of aggressive young onset breast cancer. It underscores the need to integrate comprehensive cancer risk assessment and genomic testing in the management of newly diagnosed Black women with breast cancer across the African Diaspora, enabling improved cancer control in admixed underserved and understudied populations.

**Supplementary Information:**

The online version contains supplementary material available at 10.1007/s10549-022-06560-0.

## Introduction

Breast cancer is the most commonly diagnosed cancer among women worldwide [1]. This non-communicable disease is the leading cause of female deaths in many countries, with widening disparities in outcomes among developed and developing countries. Geographic differences in incidence and mortality are due to many intrinsic (e.g., genetic) and extrinsic (e.g., environment, lifestyle) factors [2]. Despite notable differences, “one-size-fits-all” cancer control strategies are usually applied in screening and treatment within and across countries, which have led to widening disparities in breast cancer mortality and morbidity among different racial/ethnic groups [3].

This rising global burden of breast cancer in low- to middle-income countries demands innovative interventions to accelerate progress in cancer control and prevention. Through genomic analysis of breast cancer predisposition genes, the burden of inherited susceptibility to breast cancer in diverse populations can be better estimated, allowing clinical management and treatment recommendations to be tailored to the needs of high-risk women and their families [4]. With advances in high-throughput sequencing technologies, it is now possible to analyze numerous genomic regions simultaneously at greatly reduced cost. Many multi-gene panels such as the BROCA panel have been developed and applied successfully in large genetic testing studies in the United States and Europe [5, 6]. We previously reported the high prevalence of highly penetrant pathogenic variants in *BRCA1*, *BRCA2*, *PALB2*, and *TP53* genes in consecutive women presenting with advanced breast cancer at tertiary hospitals in Nigeria, Cameroon, and Uganda [7, 8].

Black women across the African Diaspora have the worst outcomes from breast cancer of all ethnic/racial groups. Given the reported high prevalence of aggressive breast cancer in young Brazilian women [9], we sought to examine the burden of inherited breast cancer in a convenient sample of consecutive women with breast cancer ascertained in a cancer risk clinic in the State of Bahia in the Northeast region of Brazil. This region has a large population of African descendants as it remains segregated and is primarily inhabited by former descendants of slaves. The African ancestral proportion revealed through genomic admixture studies is the highest in this region in comparison with other regions of Brazil [10].

## Methods

### Study population and eligibility

Between 2008 and 2015, we recruited women with breast cancer referred by their primary care physicians to the Cancer Risk Assessment Program of the Serviço de Oncogenética of Laboratório de Imunologia e Biologia Molecular (ICS-UFBA). This public laboratory service is part of the Brazilian National Network of Hereditary Cancer. Women with breast cancer are usually referred to this service from private and public clinics and hospitals. Since this service is provided by a public entity, counseling was free of charge and more than 90% of patients were willing to participate in the research. To develop a reference control panel to improve interpretation of our findings, we recruited a cohort of cancer-free women who are not the relatives of the cases but were undergoing routine laboratory tests (for regular clinical checkups or for an evaluation of other diseases) in the same laboratory between 2014 and 2015.

All participants signed informed consents, and data regarding their epidemiological and clinical profiles were collected along with questionnaires administered by a research coordinator. The research protocol #1.383.884 was approved by the Brazilian National Committee of Ethics in Research (CONEP, Comissão Nacional de Ética em Pesquisa), the University of Chicago, and the University of Washington, where the sequencing was performed.

### Next-generation sequencing and genomic analysis

The genomic DNA was extracted from peripheral blood using a commercial kit, the DNeasy® blood and tissue kit (QIAGEN, German). The quality and quantity of the genomic DNA was assessed with 2% agarose gel electrophoresis analysis and the Quant-iT™ PicoGreen™ dsDNA Assay Kit (Invitrogen, Thermo Scientific, USA). A total of 28 susceptibility genes were analyzed in the BROCA panel. Genes sequenced included established breast cancer genes of both high and moderate penetrance, and genes that have been suggested as candidate breast cancer genes, with varying levels of evidence: *ATM, ATR, BAP1, BARD1, BRCA1, BRCA2, BRIP1, CDH1, CHEK1, CHEK2, CTNNA1, FAM175A, FANCM, GEN1, MRE11A, NBN, PALB2, PPM1D, PTEN, RAD51B, RAD51C, RAD51D, RECQL, RINT1, SLX4, STK11, TP53,* and *XRCC2*.

Paired-end reads were mapped to the human genome reference hg19. Subsequently, single-nucleotide variants and small insertions and deletions were called as previously described in detail [5, 11], and copy number variants were detected as well [12]. Only variants that led to a loss of gene function or were experimentally demonstrated to damage gene function were included in further analyses. Interpretations of possible splice variants were based on in silico algorithms or on experimental results from our own work or that of others.

### Statistical analysis

Descriptive analysis was performed using Epi Info™ software (CDC, Atlanta, GA, USA) and SPSS® (SPSS Inc. Chicago, IL, USA). Given the limited sample size of the study, we performed an exploratory analysis to estimate performance of different breast cancer risk assessment tools using online calculators: the Myriad Risk calculator [13], the PENN II Risk Model [14], and BRACAPRO [15], based on the *BRCA1* and *BRCA2* mutational profiles and clinical and epidemiological data.

## Results

### Clinical characteristics

Over 90% of the study participants were from the Northeast region of Brazil, particularly from the State of Bahia. About 67.0% of the breast cancer cases self-reported as African-descended (Black), with enriched family history of cancer, including breast cancer (~ 65%) (Table 1). The mean age at breast cancer diagnosis among the cases was 44.1 ± 11.3 years while unaffected women were older, with a mean age at interview of 52.2 ± 13.6 years. The breast cancer patients were predominantly diagnosed with breast cancer only (96.5%), followed by breast and ovarian (2.3%) (Table 1). Invasive ductal carcinoma was the most common diagnosis in breast cancer patients of both African and non-African ancestry, 81.9% and 79%, respectively. The tumor subtypes were classified by immunohistochemistry for expression of hormone receptors (estrogen receptor [ER] and progesterone receptor [PR]) and human epidermal growth factor receptor 2 (HER2) (Table 2). If HER2 was classified as 2+ by immunohistochemistry, additional analysis was performed by using fluorescent in situ hybridization. The ER+/PR+/HER2- was the most common classification, followed by ER+/PR-/HER2-, triple-negative and HER2+ (41.6%, 16.2%, 15.5%, and 10.4% , respectively).

**Table 1:** Characteristics of Brazilian cases and unaffected volunteers

Characteristic		Cases (*n* = 173)	Unaffected volunteers (*n* = 119)
		*N* (%)	*N* (%)
Age (years)	≤ 50	131 (79.3%)	50 (42.01%)
	> 50	42 (20.7%)	69 (57.9%)
	Mean ± SD	44.1 ± 11.3	52.2 ± 13.6
Type of cancer	Breast only	167 (96.5%)	–
	Breast and ovarian	4 (2.3%)	–
	Other	2 (1.2%)	–
Self-reported ancestry	White	45 (26%)	10 (8.4%)
	African-descended	116 (67%)	108 (90.8%)
	Other	12 (6.9%)	1 (0.8%)
Family history of cancer	Yes	111 (64.2%)	47 (39.5%)
	No	62 (35.8%)	72 (60.5%)

**Table 2:** Spectrum of pathogenic variants in breast cancer susceptibility genes among Brazilian cases

ID	Variant	Type of cancer	Age at diagnosis	Self-reported ancestry	Family history of cancer	Histology	Tumor grade	Tumor stage	ER	PR	HER2
ACM076	*NBN* c.156_157delTT	Breast	56	Other	No	IDC	–	T4cN3M1	Pos	Pos	Pos
ACM088	*BRIP1* c.1741C>T	Breast	40	White	No	IDC	IV	T2N1M0	Pos	Pos	–
CM003	*BRCA2* c.2111delC	Breast	42	Other	Yes	IDC	III	T2N0M0	Pos	Neg	Neg
CM023	*BRCA1* c.3331_3334delCAAG	Breast	36	African-descended	Yes	IDC	I	T1N0M0	Pos	Pos	Neg
CM033	*BRCA1* c.1115G>A	Breast and Ovarian	36	African-descended	Yes	IDC + Lobular carcinoma (Breast); Serous adenocarcinoma (Ovarian)	IIB	T2N1M0	Pos	Pos	Neg
CM048	*BRCA1* c.211A>G	Breast	39	African-descended	Yes	Medullary carcinoma of the breast	–	–	Neg	Neg	Pos
CM130	*BRCA1* c.815_824dupAGCCATGTGG	Breast and Thyroid	64	White	Yes	IDC	–	–	Neg	Neg	Neg
CM179	*BRIP1* c.2392C>T	Breast and Ovarian	42	African-descended	Yes	Lobular carcinoma	–	–	Pos	Pos	Neg
CM211	*SLX4* c.4828delT	Breast	28	White	Yes	DCIS	III	TisN0M0	Neg	Neg	Pos
CM223	*BRCA1* c.3331_3334delCAAG	Breast	28	African-descended	Yes	IDC	III	T2N0M1	Neg	Neg	Neg
CM242	*BRCA1* c.211A>G	Breast	41	African-descended	Yes	Medullary carcinoma of the breast	–	–	Neg	Neg	Neg
CM247	*BRCA2* c.5904_5907delAGTC	Breast	34	African-descended	Yes	IDC	II	T2N1M0	Pos	Neg	Neg
CM252	*BRCA1* c.211A>G	Breast	52	African-descended	Yes	IDC	IIa	T2N0M0	Neg	Neg	Neg
CM266	*PALB2* c.1671_1674delTATT	Breast	35	African-descended	Yes	IDC	–	–	Pos	Pos	Neg
CM277	*PALB2* c.1671_1674delTATT	Breast	49	African-descended	No	IDC	II	–	Pos	Pos	Neg
CM278	*BRIP1* c.2097+1G>C	Breast	52	White	Yes	IDC	II	pT2pN0pMx	Neg	Neg	Pos
CM293	*PALB2* c.355delC	Breast	49	White	Yes	IDC	–	–	Pos	Pos	–
CM297	*ATM* c.3801delG	Breast	38	African-descended	Yes	IDC	–	–	Pos	Pos	Pos
CM309	*FANCM* c.5766_5769delGACT	Breast	38	African-descended	Yes	IDC	–	–	Pos	Pos	Neg
CM318	*BRCA2* c.7672G>T	Breast	27	African-descended	Yes	DCIS	IV	T3N2M1	Pos	Pos	Pos
CM322	*FAM175A* c.1011delA	Breast	43	African-descended	No	IDC	III	pT4pN3	Pos	Neg	Pos
CM362	*BRCA2* c.1389_1390delAG	Breast	47	African-descended	No	IDC	II	–	Pos	Pos	Neg
CM385	*TP53* c.1010G>A	Breast	28	African-descended	Yes	IDC	II	T1N1M0	Pos	Pos	Neg
CM389	*BRCA1* c.1327A>T	Breast	34	African-descended	Yes	IDC	–	–	Neg	Neg	Neg
CM403	*BRCA2* c.8488-1G>A	Breast	34	African-descended	Yes	IDC	II	T1N0M0	Pos	Neg	Neg
CM420	*BRCA1* c.3331_3334delCAAG	Breast	38	African-descended	Yes	IDC	II	T2N0M0	–	–	–
CM440	*BRCA2* c.736delT	Breast	42	African-descended	Yes	IDC	IV	T4cN2MX	Pos	Pos	Pos
CM512	*ATM* c.7913G>A	Breast	38	African-descended	Yes	IDC	II	T1N1M0	Pos	Pos	Neg
CM243	*BRCA1* c.470_471delCT	Breast	60	White	Yes	IDC	–	–	Neg	Neg	Neg
CM536	*BRCA2* c.1389_1390delAG	Breast	46	African-descended	Yes	IDC	II	T1N0M0	Neg	Pos	Neg
CM550	*BRCA1* c.3331_3334delCAAG	Breast	26	African-descended	No	IDC	II	pT3pN3a	Neg	Neg	Pos
CM564	*BRCA1* c.5251C>T	Breast	40	African-descended	No	IDC	II	–	Neg	Neg	Neg
CM575	*BRCA2* c.3860delA, *BARD1* c.1921C>T	Breast	41	African-descended	Yes	IDC	–	–	–	–	–
CM581	*BRCA2* c.6938-1G>C	Breast	43	African-descended	Yes	IDC	II	T2N0M0	Pos	Pos	Neg
CM582	*BRCA2* c.2T>G	Breast and Ovarian	49	African-descended	No	IDC (Breast) and Serous papillary cystadenocarcinoma (Ovarian)	Ib (breast) / III (ovarian)	T2N0M0	–	–	–
CM590	*ATM* c.8264_8268delATAAG	Breast	41	African-descended	Yes	–	–	–	–	–	–

*ER* estrogen receptor, *DCIS* ductal carcinoma in situ, *IDC* invasive ductal carcinoma, *HER2* human epidermal growth factor receptor 2, *Pos* positive, *PR* progesterone receptor, *Neg* negative

### Spectrum of pathogenic variants in breast cancer susceptibility genes

**Fig. 1:**
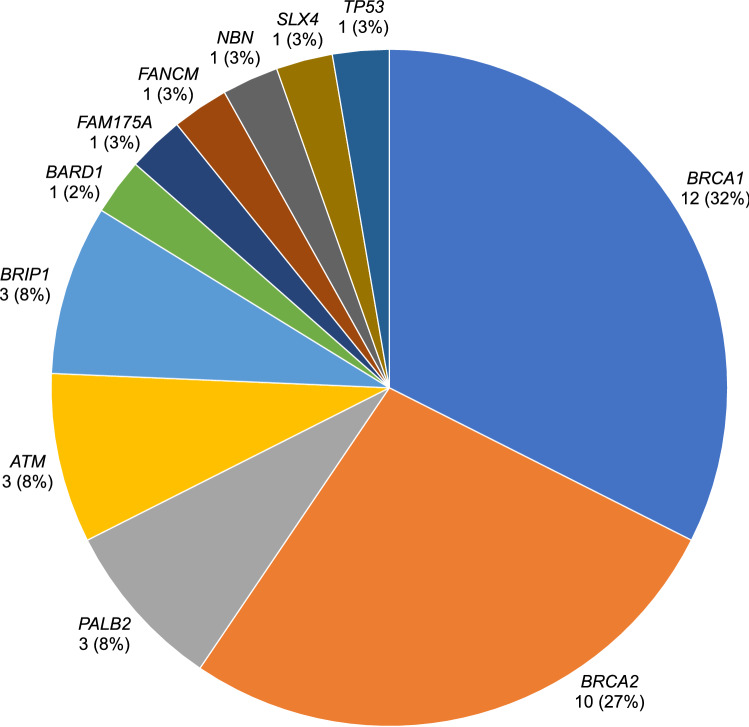
Genes with pathogenic variants in breast cancer patients from the Northeast region of Brazil. A total of 37 pathogenic variants were identified. Number and proportion of pathogenic variant(s) in each gene are shown.

Thirty-seven loss-of-function variants (30 distinct variants) were found in 36 breast cancer patients, one of whom carried both *BARD1*:c.1921C>T and *BRCA2*:c.3860delA (Fig. 1, Table 2 and Supplementary Table 1). In the cohort of cancer-free women, only one individual carried a pathogenic variant, *RAD51C*:c.264_265insA. Among self-reported African-descended breast cancer patients, 24.1% (28 of 116) carried 29 pathogenic variants in *ATM, BARD1, BRCA1, BRCA2, BRIP1, FAM175A, FANCM, PALB2* and *TP53* genes. The majority of pathogenic variants were found in *BRCA1* and *BRCA2* (65.5%, 19 of 29). Four recurrent loss-of-function variants were detected in 11 African-descended breast cancer cases, *BRCA1*:c.3331_3334delCAAG, *BRCA1*:c.211A>G, *BRCA2*:c.1389_1390delAG and *PALB2*:c.1671_1674delTATT (Table 2). Pathogenic variants were found in *BRCA1, BRIP1, NBN, PALB2*, and *SLX4* genes among eight cases of non-African ancestry (14.0%, 8 of 57), with *BRCA1* and *BRIP1* being the most commonly mutated genes (50%, 4 of 8) (Table 2).

Congruous with previously published annotations, we did not observe any African-specific pathogenic variants, and the majority of tumors arising in *BRCA1* and *BRCA2* carriers were HR- and HR+, respectively [16] (Supplementary Fig. 1). In Supplementary Fig. 2, we describe in detail the *BRCA1* and *BRCA2* mutational spectrum of the study population compared with other Black women across the African Diaspora and White women in the literature [17–22].

Using different risk models for pathogenic *BRCA1* and *BRCA2*, we observed that the predicted risk was higher in breast cancer cases carrying germline loss-of-function variants in *BRCA1* and/or *BRCA2* than in other breast cancer genes, or those carrying wild-type genes (Supplementary Table 2). The data revealed that these risk models can discriminate high-risk from low-risk women (Supplementary Fig. 3), supporting the use of these models in this population.

## Discussion

Little is known about the genetic susceptibility to breast cancer of African-descended Brazilian women, an understudied population. As there is a high degree of genetic admixture among Brazilian populations [23–25], we sought to study the genetic susceptibility to breast cancer in one of the largest African-descended populations in Latin America, the inhabitants of the State of Bahia in the Northeast region of Brazil.

Using the validated BROCA panel, we identified 36 distinct pathogenic variants in 30 patients with breast cancer cases and only one pathogenic variant in cancer-free women. As observed in previous studies worldwide [4, 22, 26], *BRCA1* and *BRCA2* were the most frequently mutated genes in breast cancer patients, and their mutation frequencies in women from the Northeast region of Brazil are closer to that in Africans than the reported frequencies in African-Americans and women of European ancestry (Table 3). We compared our results with the *BRCA1* and *BRCA2* mutational spectrum reported by the Brazilian Consortium of Hereditary Cancer [18], as well as other studies [17, 19–22]. Among sixteen *BRCA1* and *BRCA2* pathogenic variants detected in African-descended breast cancer patients in this study, twelve were documented globally, five were found in self-reported African ancestry individuals in non-African countries/regions, one was found in self-reported African ancestry individuals in African countries, eight were previously reported among Brazilians, and one was newly identified (Supplementary Fig. 2). Our observation that the *BRCA1/2* pathogenic variants found in Blacks in Northern Brazil were not unique to the population agrees with the finding of a previous study by Friebel et al. [21]. *BRCA1/2* pathogenic variants in Africans are allelic heterogeneous with low frequencies [7, 8, 27]; however, the Friebel et al*.* study showed that variants identified in Africans were also reported in non-African populations [21]. The possible explanations could be: (1) to date, breast cancer mutation surveys have been done primarily in individuals of European ancestry, which increases the probability of detecting variants also found in African populations; (2) the “out of Africa” theory of early human migrations and the diversity in the African Diaspora [27] suggest that some variants found in non-African populations could be of African origin; and (3) some African-specific variants in highly admixed populations like Brazilians might not be captured due to the limited sample size of the present study.

**Table 3:** Comparison of the high-risk mutational profile among Black women across the African Diaspora and White women

Study population	Brazilian (this study)		Nigerian (Ref. [7])		Cameroonian and Ugandan (Ref. [8])		African American (Ref. [22])		CARRIERS (Ref. [26])	
Case/control	Case	Unaffected volunteers	Case	Control	Case	Control	Case	Control	Case	Control
Number of individuals	173 (67% Black)	119 (90.8% Black)	1,136	997	196	185	5,054	4,993	32,247 (78.9% White)	32,544 (76.2% White)
Ave. age (years)	44.1	52.2	47.5	47	46.2	46.6	54.4	55.2	62.1	61.2
*BRCA1*	12 (6.9%)	0	80 (7.0%)	3 (0.3%)	11 (5.6%)	2 (1.1%)	81 (1.6%)	1 (0.02%)	275 (0.9%)	37 (0.1%)
*BRCA2*	10 (5.8%)	0	47 (4.1%)	4 (0.3%)	11 (5.6%)	0	98 (1.9%)	12 (0.2%)	417 (1.3%)	78 (0.2%)
*PALB2*	3 (1.7%)	0	11 (0.4%)	0	2 (1.0%)	0	53 (1.0%)	5 (0.1%)	148 (0.5%)	38 (0.1%)
*TP53*	1 (0.6%)	0	5 (0.4%)	0	1 (0.5%)	0	5 (0.1%)	1 (0.02%)	19 (0.06%)	2 (0.01%)
Other genes	11 (6.4%)	1 (0.8%)	24 (2.1%)	11 (1.1%)	6 (3.1%)	1 (0.5%)	179 (3.5%)	95 (1.9%)	762 (2.4%)	376 (1.2%)

In this study, other genes frequently mutated were the high-risk gene *PALB2*, as well as *ATM* and *BRIP1*, each found in 1.7% of the cases. Although the prevalence of pathogenic variants in these genes varies in breast cancer patients of African ancestry [6, 7], it confirms that genes involved in DNA repair pathways are the major contributors to inherited breast cancer. Thus, their critical role in understudied populations with high burden of young onset breast cancer deserves further examination. In the South and Southeast regions of Brazil, *TP53* is the third gene most frequently mutated among breast cancer patients. Among these patients, most carry *TP53*:c.1010G>A, which has a known founder effect in those regions of Brazil. However, this pathogenic variant was quite rare (0.6%, 1 of 173) among the breast cancer cases from the Northeast region of Brazil in our study (Fig. 1 and Table 2).

The spectrum of founder mutations found in our cohort reflects the degree of ancestral admixture within the State of Bahia, where there is a significant history of immigration from Spain, Central Europe, and West Africa [23, 28, 29]. Four recurrent variants were discovered among unrelated African-descended breast cancer patients: *BRCA1*:c.3331_3334delCAAG, *BRCA1*:c.211A>G, *BRCA2*:c.1389_1390delAG, and *PALB2*:c.1671_1674delTATT. The first two were previously described in Spanish descendants [30, 31] and in the Northeast Brazilian population [29]. *BRCA2*:c.1389_1390delAG was described in diverse populations in Central Europe [32–34], while *PALB2*:c.1671_1674delTATT remains uncharacterized and was documented only twice in ClinVar. To confirm whether this *PALB2* pathogenic variant has a founder effect, further studies are needed to evaluate the carriers’ haplotypes. In addition, we observed variants that are recurrent in African populations: *ATM*:c.7913G>A [35, 36], *BRCA1*:c.815_824dupAGCCATGTGG [37, 38] and *FAM175A*:c.1011delA [39]. In addition, in the present study, cancer risk assessment tools like Myriad Risk, PENN II Risk, and BRCAPRO demonstrated an overall moderate efficiency at detecting high-risk individuals (Supplementary Table 2 and Supplementary Fig. 3). Larger population studies are needed to validate our findings to further improve the utility of risk prediction models in diverse populations.

This study has several limitations. First, the sample size is relatively small, which limits the study’s power to detect variants with lower frequencies. Second, the recruitment of the participants was clinic based and therefore may not represent true population frequencies. Lastly, the BROCA gene panel was not designed to include ancestry informative markers in the assay; therefore, we were unable to perform genetic ancestry analysis. An advanced targeted gene panel with ancestry informative markers included, or accompanied with a genotyping assaying capturing that information, may improve the risk assessment based on a person's ethnic background.

In summary, this study sought to investigate whether the high mutation rates observed in Nigeria, Cameroon, and Uganda are also present in Brazilian women of African ancestry using a validated multi-gene panel. As costs of genomic testing continues to drop, this study provides additional evidence in support of broader access to genetic testing for previously underserved and understudied Black women at high risk of young onset and aggressive forms of breast cancer. The fact that one in five patients carried a loss-of-function variant in *BRCA1*, *BRCA2*, or another breast cancer gene with a highly heterogeneous mutational spectrum underscores the importance of utilizing next-generation sequencing-based testing to develop screening and risk-reducing strategies in Northeast Brazil.

## Supplementary Information

Below is the link to the electronic supplementary material.

Supplementary file1 (EPS 80 kb) **Supplementary Figure 1.**Breast cancer subtype distribution according to genetic mutational profile in BROCA panel. Breast cancer subtypes: HR-/HER2-, HR-/HER2+, HR+/HER2-, HR+/HER2+, HR-/HER2?, HR+/HER2?, and Missing. HER2, human epidermal growth factor receptor 2; HER2?, HER2 status unknown; HR, hormone receptor.

Supplementary file2 (EPS 40 kb) **Supplementary Figure 2. ***BRCA1* and *BRCA2* mutational spectrum in breast cancer cases from the Northeast region of Brazil and other populations worldwide. SRAA, self-reported African ancestry.

Supplementary file3 (EPS 70 kb) **Supplementary Figure 3.** Performance of risk prediction models. We divided our study participants into three genomically stratified groups: high-risk cases (who carried pathogenic variants in breast cancer genes), low-risk cases (who did not carry any pathogenic variants), and cancer-free women. (a) The values of AUC of the risk prediction tools improved slightly for discriminating the high-risk cases from low-risk patients. (b) When the high-risk group was compared with low-risk group and cancer-free women, the power of the risk models to discriminate high-risk individuals from low-risk was increased.

Supplementary file4 (DOCX 34 kb)

Supplementary file5 (DOCX 31 kb)

## Data Availability

All data described and analyzed here are available upon request.
