# Supplementary material for: Mutational spectrum of breast cancer susceptibility genes among women ascertained in a cancer risk clinic in Northeast Brazil
Source: Breast Cancer Res Treat. 2022 Mar 30;193(2):485–94. doi: 10.1007/s10549-022-06560-0 (PMC9090684; doi:10.1007/s10549-022-06560-0)
Supplement: Supplementary file 4 — Supplementary file4 (DOCX 34 kb) [file 10549_2022_6560_MOESM4_ESM.docx]

**Supplementary Table 1.** Annotation of the loss-of-function variants detected in a cohort of African-descended Brazilians.

| **Gene** | **Transcript** | **Coding impact** | **HGVS** | |
| --- | --- | --- | --- | --- |
|  |  |  | **Coding** | **Protein** |
| ***ATM*** | NM_000051.3 | Frameshift | c.3802delG | p.Glu1267_Val1268insTer |
| ***ATM*** | NM_000051.3 | Nonsense | c.7913G>A | p.Trp2638Ter |
| ***ATM*** | [NM_000051.3](http://www.ncbi.nlm.nih.gov/nuccore/NM_000051.3) | Frameshift | c.8264_8268delATAAG | p.Tyr2755CysfsTer12 |
| ***BARD1*** | NM_000465.3 | Nonsense | c.1921C>T | p.Arg641Ter |
| ***BRCA1*** | NM_007294.3 | Nonsense | c.5251C>T | p.Arg1772Ter |
| ***BRCA1*** | NM_007294.3 | Frameshift | c.3331_3334delCAAG | p.Gln1111AsnfsTer5 |
| ***BRCA1*** | NM_007294.3 | Nonsense | c.1327A>T | p.Lys443Ter |
| ***BRCA1*** | NM_007294.3 | Nonsense | c.1115G>A | p.Trp372Ter |
| ***BRCA1*** | NM_007294.3 | Frameshift | c.815_824dupAGCCATGTGG | p.Thr276AlafsTer14 |
| ***BRCA1*** | NM_007294.3 | Frameshift | c.470_471delCT | p.Leu156_Ser157insTer |
| ***BRCA1*** | NM_007294.3 | Missense | c.211A>G | p.Arg71Gly |
| ***BRCA2*** | NM_000059.3 | Missense | c.2T>G | p.Met1Arg |
| ***BRCA2*** | NM_000059.3 | Frameshift | c.738delT | p.Phe246LeufsTer5 |
| ***BRCA2*** | NM_000059.3 | Frameshift | c.1389_1390delAG | p.Val464GlyfsTer3 |
| ***BRCA2*** | NM_000059.3 | Frameshift | c.2111delC | p.Pro704GlnfsTer26 |
| ***BRCA2*** | NM_000059.3 | Frameshift | c.3860delA | p.Asn1287IlefsTer6 |
| ***BRCA2*** | NM_000059.3 | Frameshift | c.5904_5907delAGTC | p.Val1969HisfsTer34 |
| ***BRCA2*** | [NM_000059.3](http://www.ncbi.nlm.nih.gov/nuccore/NM_000059.3) | Intronic | c.6938-1G>C | - |
| ***BRCA2*** | NM_000059.3 | Nonsense | c.7672G>T | p.Glu2558Ter |
| ***BRCA2*** | [NM_000059.3](http://www.ncbi.nlm.nih.gov/nuccore/NM_000059.3) | Intronic | c.8488-1G>A | - |
| ***BRIP1*** | NM_032043.2 | Nonsense | c.2392C>T | p.Arg798Ter |
| ***BRIP1*** | NM_032043.2 | Intronic | c.2097+1G>C | - |
| ***BRIP1*** | NM_032043.2 | Nonsense | c.1741C>T | p.Arg581Ter |
| ***FAM175A*** | NM_139076.2 | Frameshift | c.1011delA | p.Ala338LeufsTer12 |
| ***FANCM*** | NM_020937.3 | Frameshift | c.5766_5769delGACT | p.Thr1923ProfsTer2 |
| ***NBN*** | NM_002485.4 | Frameshift | c.156_157delTT | p.Ser53CysfsTer9 |
| ***PALB2*** | NM_024675.3 | Frameshift | c.1671_1674delTATT | p.Ile558LysfsTer2 |
| ***PALB2*** | NM_024675.3 | Frameshift | c.355delC | p.Gln119LysfsTer58 |
| ***RAD51C*** | NM_058216.2 | Frameshift | c.264_265insA | p.Glu89ArgfsTer4 |
| ***SLX4*** | NM_032444.2 | Frameshift | c.4828delT | p.Ser1610ProfsTer105 |
| ***TP53*** | NM_000546.5 | Missense | c.1010G>A | p.Arg337His |
