# Supplementary material for: Mutational spectrum of breast cancer susceptibility genes among women ascertained in a cancer risk clinic in Northeast Brazil
Source: Breast Cancer Res Treat. 2022 Mar 30;193(2):485–94. doi: 10.1007/s10549-022-06560-0 (PMC9090684; doi:10.1007/s10549-022-06560-0)
Supplement: Supplementary file 5 — Supplementary file5 (DOCX 31 kb) [file 10549_2022_6560_MOESM5_ESM.docx]

**Supplementary Table 2.** Mean mutational risk estimated in breast cancer cases according to genetic susceptibility profile.

| **Risk prediction tool** | **Breast cancer susceptibility gene mutated** | | | | **P value^**^** |
| --- | --- | --- | --- | --- | --- |
|  | ***BRCA1*** | ***BRCA2*** | **Other breast cancer genes^*^** | **None** |  |
| **Myriad** | 25.2 | 26.6 | 13.1 | 10.4 | 0.004 |
| **PENN BRCA1** | 52.7 | 37.5 | 18.5 | 18.0 | 0.011 |
| **PENN BRCA2** | 23.5 | 16.5 | 16.5 | 16.0 | 0.076 |
| **BRCAPRO 1** | 71.5 | 19.4 | 7.4 | 5.6 | 0.30 |
| **BRCAPRO 2** | 12.8 | 23.2 | 6.2 | 4.2 | 0.009 |

***Other breast cancer genes*: ATM, BARD1, BRIP1, FAM175A, FANCM, NBN*, *PALB2, SLX4 and TP53*.

****Kruskal-Wallis test
